# Supplementary figures and images for: Efficient immortalization of human dental pulp stem cells with expression of cell cycle regulators with the intact chromosomal condition
Source: PLoS One. 2020 Mar 2;15(3):e0229996. doi: 10.1371/journal.pone.0229996 (PMC7051082; doi:10.1371/journal.pone.0229996)

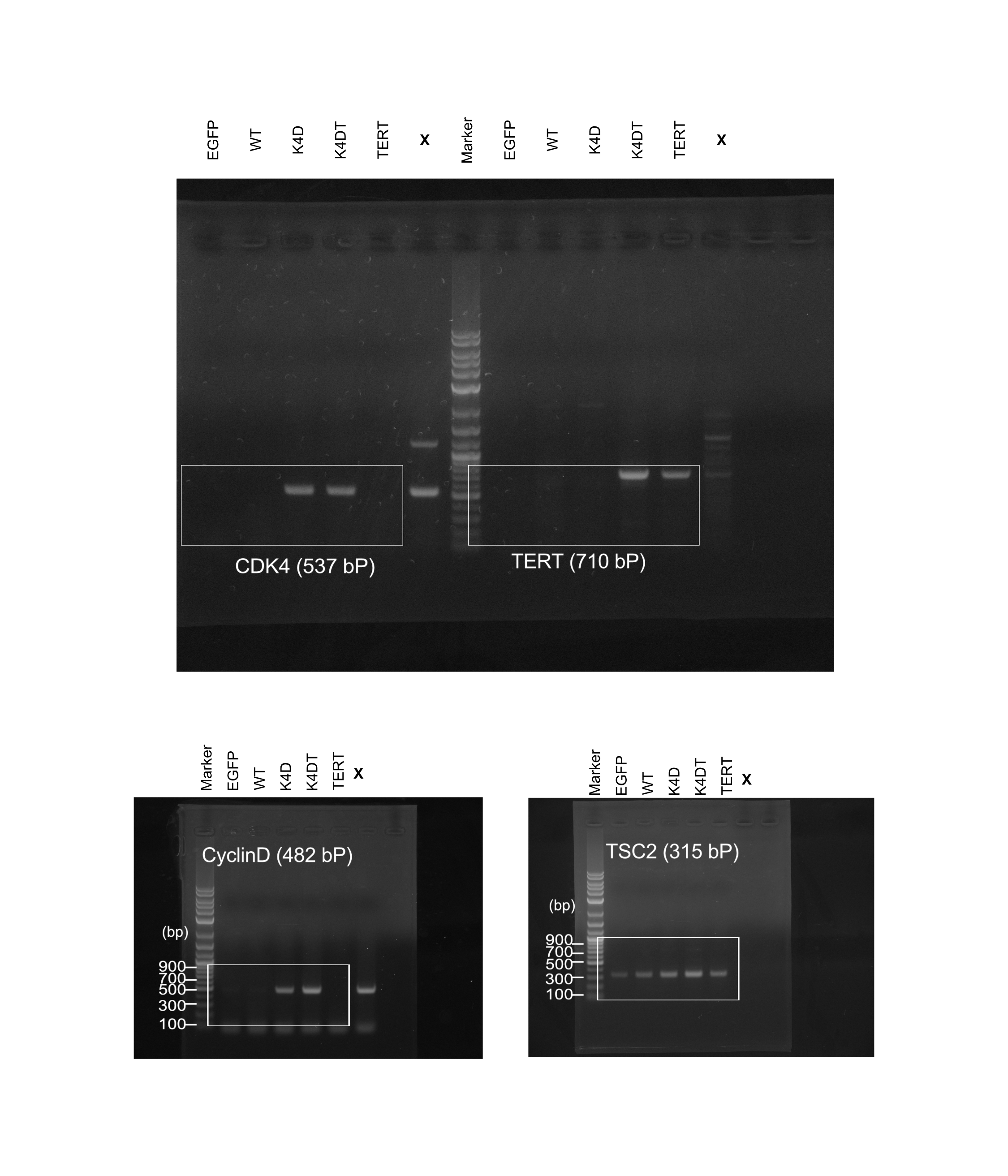

Supplement: S1 Fig — The corresponding area of the gel images were indicated by white rectangle. (TIFF) [file pone.0229996.s001.tiff]

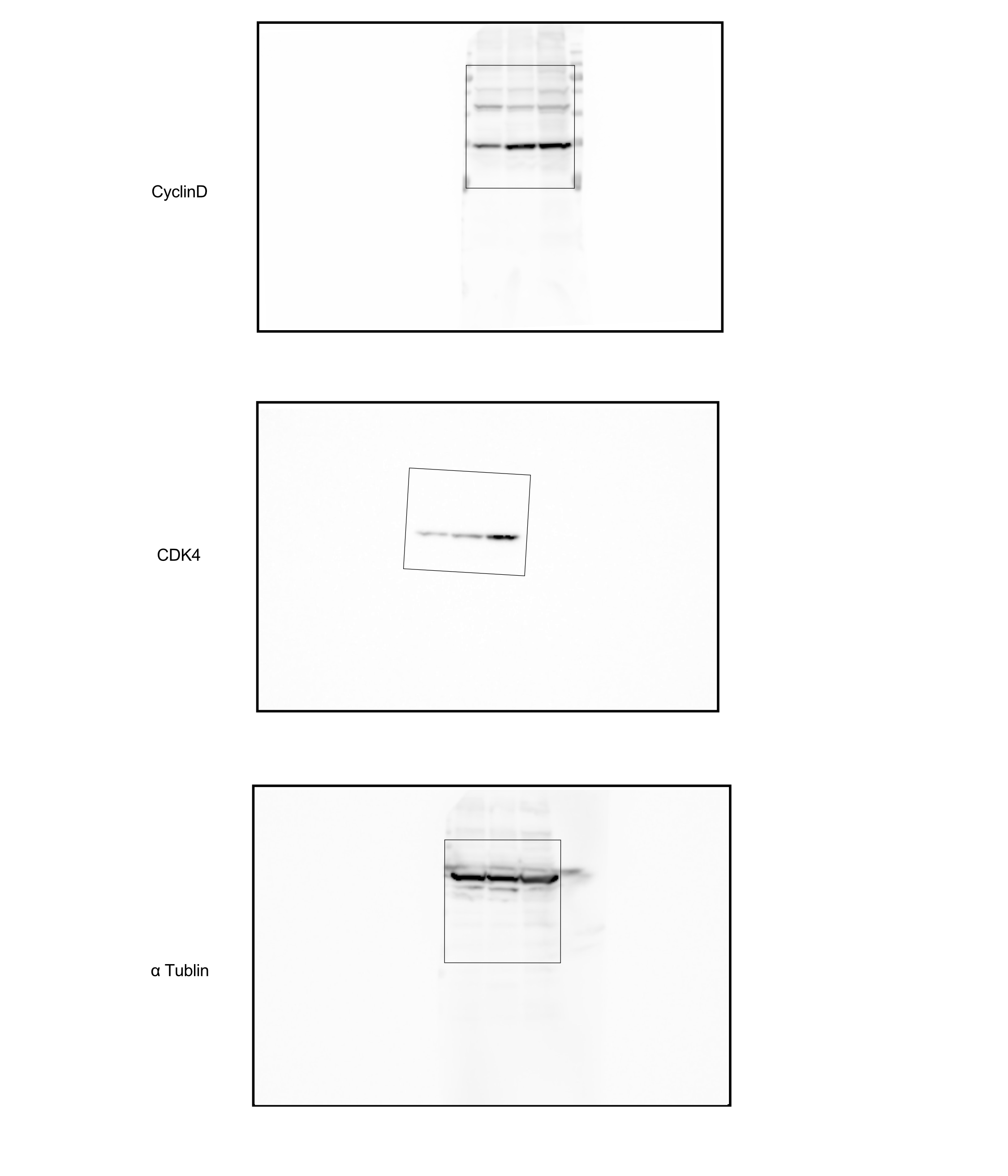

Supplement: S2 Fig — The corresponding area of the gel images were indicated by white rectangle. (TIFF) [file pone.0229996.s002.tiff]
