# Supplementary material for: Efficient immortalization of human dental pulp stem cells with expression of cell cycle regulators with the intact chromosomal condition
Source: PLoS One. 2020 Mar 2;15(3):e0229996. doi: 10.1371/journal.pone.0229996 (PMC7051082; doi:10.1371/journal.pone.0229996)
Supplement: Attachment — Submitted filename: Revision summary plos 021020 3_.doc [file pone.0229996.s003.doc]

**Revision summary**

We would like to submit our revised version of our manuscript, which entitled “Efficient immortalization of human dental pulp stem cells with expression of cell cycle regulators with the intact chromosomal condition”. All comments obtained from the reviewers were quite productive to improve our manuscript. We will list all comments and corresponding change listed below with point by point style. Furthermore, we will submit our revised manuscript with changes highlighted. In this round of revisions, we performed flow cytometric analysis and the differentiation experiments to test the characteristic of our generated cell line. We showed that our established immortalized human dental pulp stem cells preserved the expression of stemness associated markers and their differentiation properties even after passages. As we carried out our best efforts to satisfy the comments, we are glad to finish up the additional task, if it is essential for the acceptance of the manuscript.

**Journal Requirements:**

**When submitting your revision, we need you to address these additional requirements.**

**1. Please ensure that your manuscript meets PLOS ONE's style requirements, including those for file naming. The PLOS ONE style templates can be found at http://www.plosone.org/attachments/PLOSOne_formatting_sample_main_body.pdf and http://www.plosone.org/attachments/PLOSOne_formatting_sample_title_authors_affiliations.pdf**

We performed double-check the formatting style of the PLoS One, and re-formatted the entire manuscript. Now, we hope that our modification of the manuscript style would be satisfactory to the comments.

**2. In your Data Availability statement, you have not specified where the minimal data set underlying the results described in your manuscript can be found. PLOS defines a study's minimal data set as the underlying data used to reach the conclusions drawn in the manuscript and any additional data required to replicate the reported study findings in their entirety. All PLOS journals require that the minimal data set be made fully available. For more information about our data policy, please see http://journals.plos.org/plosone/s/data-availability.**

**Upon re-submitting your revised manuscript, please upload your study’s minimal underlying data set as either Supporting Information files or to a stable, public repository and include the relevant URLs, DOIs, or accession numbers within your revised cover letter. For a list of acceptable repositories, please see http://journals.plos.org/plosone/s/data-availability#loc-recommended-repositories. Any potentially identifying patient information must be fully anonymized.**

**Important: If there are ethical or legal restrictions to sharing your data publicly, please explain these restrictions in detail. Please see our guidelines for more information on what we consider unacceptable restrictions to publicly sharing data: http://journals.plos.org/plosone/s/data-availability#loc-unacceptable-data-access-restrictions. Note that it is not acceptable for the authors to be the sole named individuals responsible for ensuring data access.**

**We will update your Data Availability statement to reflect the information you provide in your cover letter.**

Based on the instruction, we included all experimental data used to the conclusions drawn in our manuscript. The data upload would be satisfactory to the comments from the journal office.

**3. PLOS ONE now requires that authors provide the original uncropped and unadjusted images underlying all blot or gel results reported in a submission’s figures or Supporting Information files. This policy and the journal’s other requirements for blot/gel reporting and figure preparation are described in detail at https://journals.plos.org/plosone/s/figures#loc-blot-and-gel-reporting-requirements and https://journals.plos.org/plosone/s/figures#loc-preparing-figures-from-image-files. When you submit your revised manuscript, please ensure that your figures adhere fully to these guidelines and provide the original underlying images for all blot or gel data reported in your submission. See the following link for instructions on providing the original image data: https://journals.plos.org/plosone/s/figures#loc-original-images-for-blots-and-gels.**

**In your cover letter, please note whether your blot/gel image data are in Supporting Information or posted at a public data repository, provide the repository URL if relevant, and provide specific details as to which raw blot/gel images, if any, are not available. Email us at plosone@plos.org if you have any questions.**

Based on the instruction, we uploaded full range blots and gel images as Supplementary Figures.

**4. PLOS requires an ORCID iD for the corresponding author in Editorial Manager on papers submitted after December 6th, 2016. Please ensure that you have an ORCID iD and that it is validated in Editorial Manager. To do this, go to ‘Update my Information’ (in the upper left-hand corner of the main menu), and click on the Fetch/Validate link next to the ORCID field. This will take you to the ORCID site and allow you to create a new iD or authenticate a pre-existing iD in Editorial Manager. Please see the following video for instructions on linking an ORCID iD to your Editorial Manager account:** [**https://www.youtube.com/watch?v=_xcclfuvtxQ**](https://www.youtube.com/watch?v=_xcclfuvtxQ)

Based on the instruction, the corresponding author create a new ORCID iD.

**5. Please ensure that you refer to Figure 5 in your text as, if accepted, production will need this reference to link the reader to the figure.**

Based on the instruction, we included Figure 5 in our manuscript to allow the reader to link the figure. Thank you for the suggestion.

**Review Comments to the Author**

**Please use the space provided to explain your answers to the questions above. You may also include additional comments for the author, including concerns about dual publication, research ethics, or publication ethics. (Please upload your review as an attachment if it exceeds 20,000 characters)**

**Reviewer #1: In this paper authors established a novel immortalized dental pulp stem cell line by co-expressing a mutant cyclin-dependent kinase 4 (CDK4 R24C ), Cyclin D1, and telomerase reverse transcriptase (TERT). They concluded that the established K4DT cell line has the advantage of being easy to handle, resulting useful in dental pulp regeneration therapy.**

**The concept is interesting, but it represents only a preliminary study. The authors should test the characteristic of the new generated cell line, such as the stemness and the ability to differentiate at least in osteogenic lineage. For these reasons, the conclusions are not supported by data.**

We appreciate very much to summarize the outline of our publication. Based on the comments, to test the characteristic of our generated cell line, termed K4DT cells, we performed flow cytometric analysis and the differentiation experiments. As a result, flow cytometry analysis of K4DT cells showed the high expression of the common stem cell markers in mesenchymal stem cell, CD90, and low expression of hematopoietic markers, CD34 and CD45, similar to the results observed in wild type cells. In the differentiation experiments, we tested the ability to differentiate in the osteogenic and adipogenic lineage. The K4DT cells were differentiated into osteoblasts immunostained with anti-osteocalcin antibody and showed marked mineralization by Alizarin Red S staining. K4DT cells also differentiated adipocytes and showed positive staining of numerous oil droplets by Oil red O.

Our data suggest that our established cell line maintained its original stemness characteristics without affecting differentiation potential.

To report the results described above, we involved new figures (Figure 7, 8, and 9 in the revised manuscript) and corresponding descriptions in the section of Materials and Methods (lines 157-183), Results (lines 248-268), and Discussion (lines 323-336).

**Reviewer #2: In this manuscript the Authors describe a new immortalized cell line obtained from dental pulp stem cells infected with retrovirus carrying the genes for a mutant cyclin-dependent kinase 4 (CDK4R24C), Cyclin D1, and telomerase reverse transcriptase (TERT). The Authors showed that such cell line have a normal karyotype and do not go in senescence after few passages. The Authors propose this cell line as a new tool to study dental pulp stem cells based regeneration protocols. The work is interesting and the experiments are clearly performed, however there are few concerns that need to be addressed a s follow:**

**In order to be used as a tool for regeneration experiments it not sufficient to prove that the cells do not go in senescence, the author should also prove that the cells keep their differentiation properties, so they should allow the cells to grow for few passages and then show that they are still able to differentiate toward at least osteogenic and adipogenic phenotype (just as a proof of concept).**

**Dental pulp stem cells have been described to express different markers (see as an example “Methods for the identification, characterization and banking of human DPSCs: current strategies and perspectives. Stem Cell Rev Rep. 2011 Sep;7(3):608-15. doi: 10.1007/s12015-011-9235-9.) the Author should show that the expression of stemness associated markers is not lost after passages.**

We appreciate very much to summarize the outline of our publication and for the critical and productive comments to our publication. Based on the suggestion, to determine whether our immortalized cells, termed K4DT cells, keep the original characteristic of dental pulp stem cells, the stemness and their differentiation abilities were assessed by using the K4DT cells at passage 8, 15, or 16. As described in the responses to the Reviewer’s comment #1, flow cytometry analysis of K4DT cells at passage 8 showed the high expression of the common stem cell markers in mesenchymal stem cell, CD90 and low expression of hematopoietic markers, CD34 and CD45, similar to the results observed in wild type cells. In addition, we studied their differentiation abilities for osteogenesis and adipogenesis by using the K4DT cells at passage15 and 16. As a result, we observed that the K4DT cells differentiated into osteoblasts and adipocytes in each differentiation medium containing differentiation inducers. K4DT cells at passages 15 after approximately 3 months that started cell culture showed positive staining of calcified materials by Alizarin red S after 17 days. The K4DT cells also differentiated into osteoblasts immunostained with anti-osteocalcin antibody after 25 days. After 23 days of culture with adipoinduction medium, the K4DT at passages 16 developed numerous lipid droplets stained with Oil Red O. These results indicated that our established immortalized human dental pulp stem cells preserved the expression of stemness associated markers and their differentiation properties even after passages.

To report the results described above, we involved new figures (Figure 7, 8, and 9 in the revised manuscript) and corresponding descriptions in the section of Materials and Methods (lines 157-183), Results (lines 248-268), and Discussion (lines 323-336).
